# Kyphectomy for severe kyphosis with pyogenic spondylitis associated with myelomeningocele: a case report

**DOI:** 10.1186/1748-7161-6-5

**Published:** 2011-04-08

**Authors:** Kenji Yoshioka, Kota Watanabe, Yoshiaki Toyama, Kazuhiro Chiba, Morio Matsumoto

**Affiliations:** 1 Department of Orthopaedic Surgery, School of Medicine Keio University, 35 Shinanomachi, Shinjuku, Tokyo 160-8582, Japan; 2 Department of Advanced Therapy for Spine and Spinal Cord Disorders, School of Medicine Keio University, 35 Shinanomachi, Shinjuku, Tokyo 160-8582, Japan

## Abstract

A 32-year-old woman was referred to our hospital for a refractory ulcer on her back. She had a history of myelomeningocele with spina bifida that was treated surgically at birth. The ulcer was located at the apex of the kyphosis. An X-ray film revealed a kyphosis of 154° between L1 and 3 and a scoliosis of 60° between T11 and L5. Computed tomography, magnetic resonance imaging and laboratory data indicated the presence of a pyogenic spondylitis at L2/3. To correct the kyphosis and remove the infected vertebrae together with the skin ulcer, kyphectomy was performed. Pedicle screws were inserted from T8 to T12 and from L4 to S1. The dural sac was transected and ligated at L2, followed by total kyphectomy of the L1-L3 vertebrae. The spinal column was reconstructed by approximating the ventral wall of the T12 vertebral body and the cranial endplate of the L4 vertebra. Postoperatively, the kyphosis was corrected to 61° and the scoliosis was corrected to 22°. In the present case, we treated the skin ulcer and pyogenic spondylitis successfully by kyphectomy, thereby, preventing recurrence of the ulcer and infection, and simultaneously obtaining sufficient correction of the spinal deformity.

## Background

Myelomeningocele is a defect of the neural tube that occurs during the first 3 to 4 weeks of human embryogenesis [1]. The pathologic features of myelomeningocele include a defect of the posterior bony elements of the spine and poor viable muscular covering, resulting in the exposure of the dural sac containing neural tissues. Eight to 28% of patients with myelomeningocele develop kyphosis [2-8], since the deficiency of posterior bony elements of the spine as well as lateral displacement of spinal extensor muscles resulted in the functional loss of spinal erection, forcing the spine into flexion [1,2,9]. The progressive kyphosis causes various disorders including respiratory insufficiency, trunk imbalance, bladder and bowel dysfunction, and refractory skin ulcers at the bony prominence of the kyphosis [1,2,5,7,8,10]. Since kyphosis progresses rapidly with skeletal growth and conservative treatments, bracing are rarely effective for preventing the progression of kyphosis, therefore, surgical treatments were performed during an infantile, juvenile or adolescent periods in the majority of cases. To our knowledge, only one adult patient (20 years old) who was surgically treated for hyperkyphosis was reported in 142 patients from ten previous articles [1-11]. We report a rare adult case of severe kyphosis associated with myelomeningocele that developed pyogenic spondylitis at the apex of kyphosis.

## Case presentation

A 32-year-old female suffering from a refractory skin ulcer on her back was referred to our hospital. She was surgically treated for myelomeningocele soon after birth and had also received a ventricle-atrial shunt at the age of 17 years and a ventricle-peritoneal shunt at the age of 19 years for hydrocephalus. She was unable to walk because of complete paraplegia below the level of T8. The ulcer had been treated twice with a musculocutaneus flap, at the ages of 11 and 31 years which failed.

**Figure 1:**
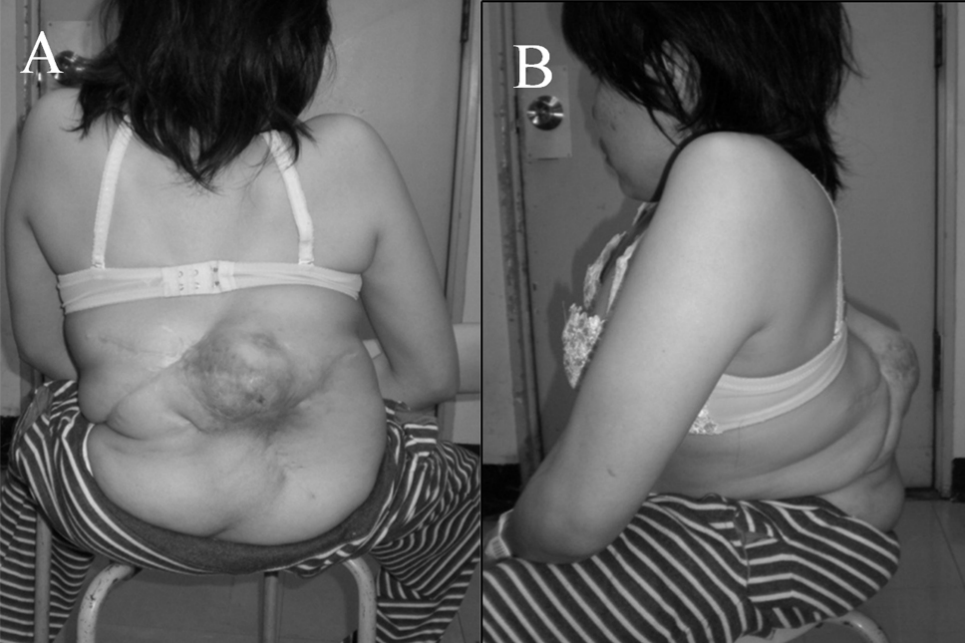
**Physical appearance**. Anterior-posterior (A-P) view (A) and lateral view (B) showing the ulcer at the bony prominence of the apex of kyphosis.

**Figure 2:**
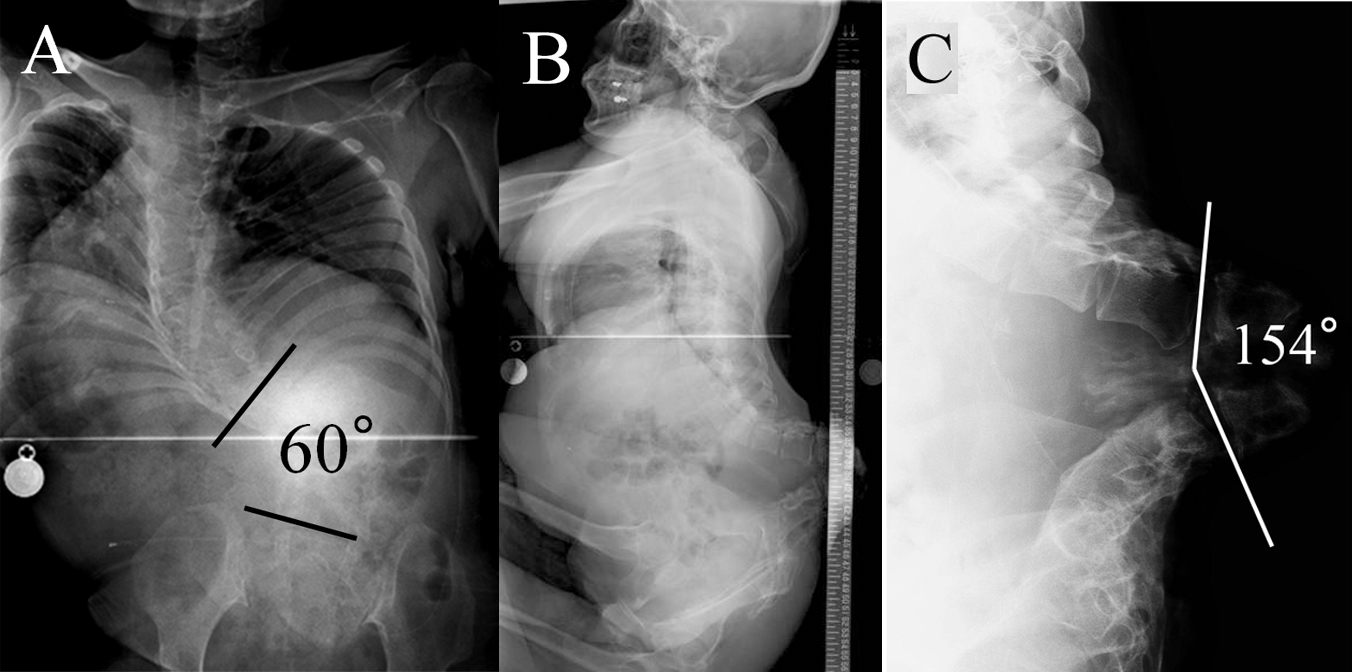
**Preoperative radiographs**. A: A-P view showing a scoliosis of 60° between T11 and L5. B: Lateral view of the whole spine showing severe kyphosis with severe thoracic lordosis and horizontally oriented pelvic. No obvious sagittal imbalance is recognized. C: Spot lateral view showing a kyphosis of 154° between L1 and 3.

**Figure 3:**
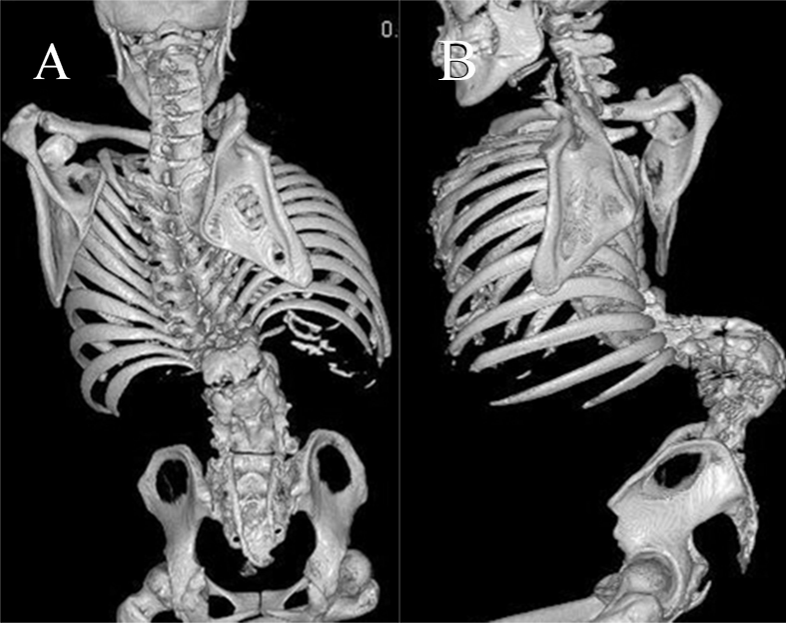
**Preoperative three dimensional CT scan**. A-P view (A) and lateral view (B) demonstrating severe kyphosis at the lumbar region and spina bifida below L1.

**Figure 4:**
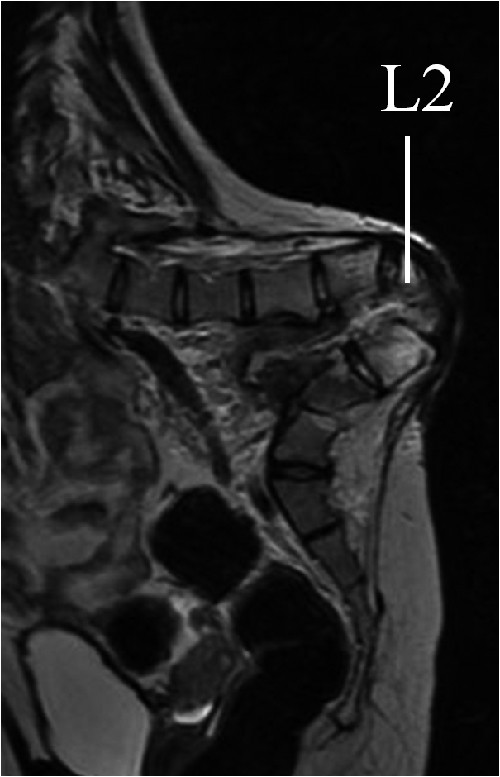
**Preoperative sagittal MR images (T2-weighted)**. An abscess formation at the L2/3 intervertebral disc and the ventral side of the L2 vertebral body is recognized.

The ulcer was located at a bony prominence of the apex of the kyphosis (Figure 1). The cultures of exudates from the ulcer were positive for Methicillin-resistant *Staphylococcus aureus *(MRSA). Laboratory data revealed an elevated serum C-reactive protein (CRP) level of 2.52 mg/dL and a white blood cell (WBC) count of 13800/μL. X-ray films showed a kyphosis of 154° between L1 and 3 and a scoliosis of 60° between T11 and L5 (Figure 2). No segmental motion at the kyphosis was recognized using anterior and posterior flexion-extention X-rays. Additionally, the flexibility of the compensatory curves in the thoracic and lumbar areas was extremely low. Computed tomography (CT) showed a wedged vertebra at L2 and defects of the laminae from L1 to the sacrum (Figure 3). Magnetic resonance imaging (MRI) revealed an abscess formation at the L2/3 intervertebral disc and the ventral side of the L2 vertebral body (Figure 4). Thus, the diagnosis of refractory ulcer with pyogenic spondylitis at the apex of the kyphosis was established.

**Figure 5:**
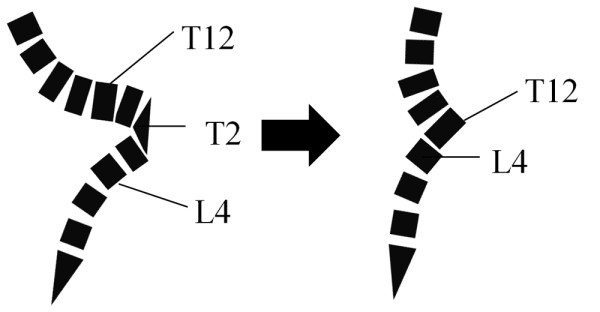
**Schematic drawing illustrating correction of kyphosis after kyphectomy**. The ventral wall of T12 is approximated to the cranial endplate of L4.

**Figure 6:**
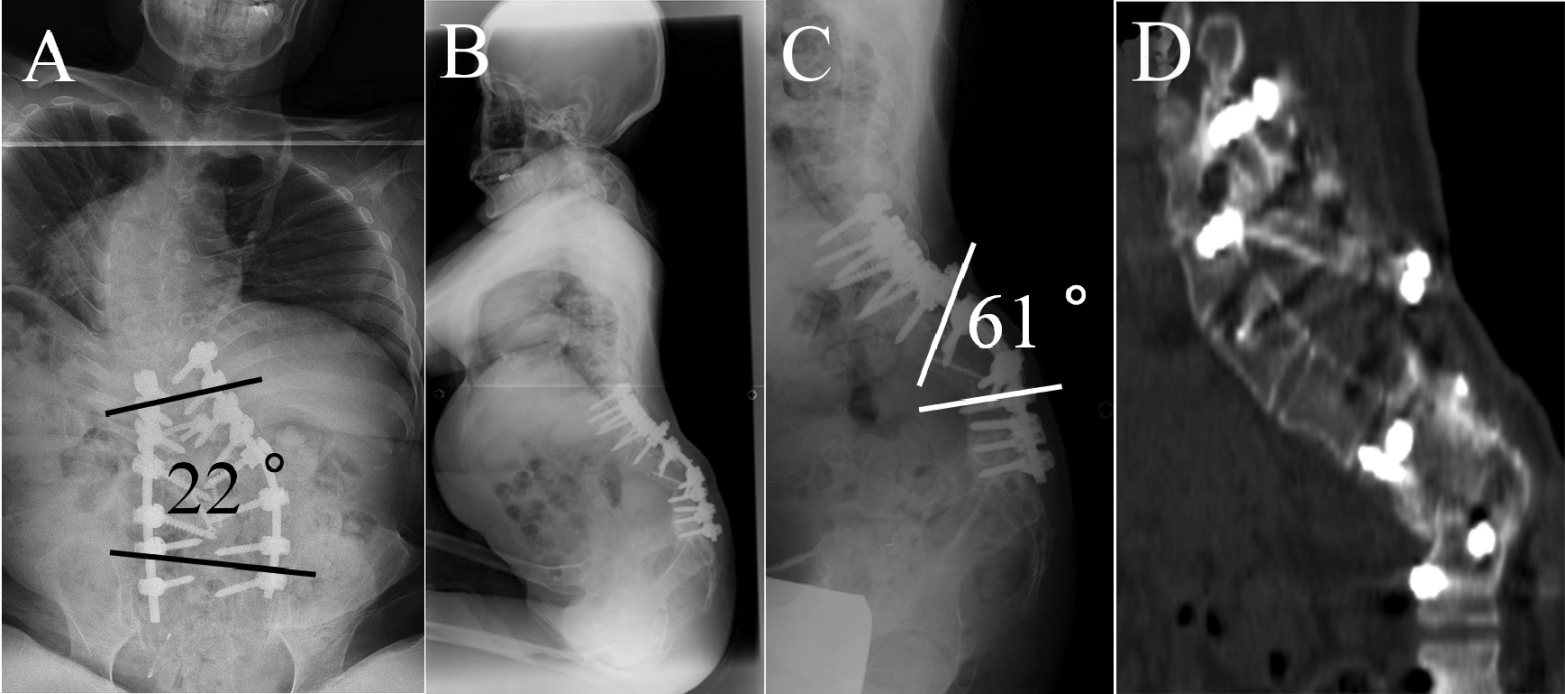
**Postoperative radiographs**. A: A-P view showing correction of scoliosis to 22°. B: Lateral view of the whole spine showing sagittal balance was well maintained and the inclination of pelvic apparently changed to more vertically oriented. C: Lateral spot view showing correction of kyphosis to 61°. D: Reconstructed sagittal CT image demonstrating solid bone fusion at the kyphectomy site.

Preoperative intravenous administration of Vancomycin^® ^for pyogenic spondylitis resulted in a decrease in the serum WBC count and CRP level to normal ranges. Subsequently, kyphectomy from L1-3 for the refractory ulcer was performed. A midline longitudinal incision was made and posterior elements of the spine and the lateral aspects of the vertebral bodies from L1to 3 were meticulously exposed. Pedicle screws were placed bilaterally from T8 to T12 and from L4 to S1. Following the ligation and transection of the dural sac at the level of L2, kyphectomy from L1 to L3 was performed. The spinal column was reconstructed by approximating the ventral wall of the T12 vertebral body and the cranial endplate of the L4 vertebra (Figure 5). In addition to the pedicle screws, supplemental fixation was added to enhance the stability of the spinal column using two cortical screws penetrating from L4 to T12. Finally, the ulcer was completely resected, and the wound was closed primarily without a musculocutaneous flap. The operative time was 305 minutes, and the intraoperative blood loss was 980 ml. After the surgery, the scoliosis was corrected to 22°, and the kyphosis was corrected to 61° with a correction rate of 63% and 61%, respectively (Figure 6A, B, C). Although sitting balance and levels of activities of daily living were maintained after surgery, another ulcer in her perineal area developed, requiring two surgical treatments with musculocutaneus flaps. Two years after the surgery, there was no recurrence of the ulcer on her back, and a solid bony fusion was confirmed on CT images without loss of correction (Figure 6D).

## Discussion

Kyphectomy for the treatment of severe kyphosis associated with myelomeningocele was first reported in 1968 by Sharrard [9]. Since then, various techniques for fixation and correction following kyphectomy have been reported. In early reports, short posterior fusions using staples, surgical wires and screws were resulted in significant correction loss in the majority of cases [4,7,12]. Thus, long fusions are now preferred after kyphectomy by many surgeons [1,2,6,8]. Kocaoğlu [12] reported that segmental pedicle screw fixation after kyphectomy was effective for achieving a good sagittal balance. Additionally, some authors have also reported that segmental pedicle screw fixation after kyphectomy is safe and effective for the treatment of kyphotic deformities in patients with achondroplasia providing strong stability [13,14].

One of the important purposes of the deformity surgery is restoring or maintaining a good sagittal balance. Especially in non-ambulatory patients, sitting without arm supports is crucial. Preoperatively, the patient could sit without any supports, although somewhat unstably. Moreover, the compensatory curves were very rigid, and maximum correction of the kyphosis could result in further sagittal imbalance deteriorating the patient's activities of daily living. Thus, ventral wall of the T12 vertebral body and cranial endplate of the L4 vertebra body were approximated to avoid excessive correction, resulting in the correction of the kyphosis from 154° to 61°, yielding a correction rate of 61%. In previous papers, higher correction rates of from 64% to 86% were reported [1,2,5,6,8]. Since most previous cases were younger than 20 years of age before skeletal maturity, the development of sagittal imbalance after maximum correction of kyphosis could be avoided because of sufficient flexibility of the upper and lower compensatory curves. On lateral X-ray (Figure 6B), though the trunk inclination was maintained, the inclination of pelvic apparently changed to a more vertical orientation. However, the sitting balance and levels of activities of daily living were maintained after surgery.

The cause of the refractory ulcer in the present case was continuous pressure at the apex of the kyphosis while the patient was sitting in a wheelchair. Additionally, chronic pyogenic spondylitis caused by MRSA at the apex of the kyphosis hinders the ulcer from healing. Although the infection was well controlled by the administration of Vancomycin^® ^prior to surgery, we were afraid of the impending sepsis induced by the residual abscess, and decided that the removal of the infected vertebra by kyphectomy was necessary to prevent the recurrence of infection and ulcer. In the present case, a kyphectomy followed by segmental pedicle screw fixation was selected to prevent the correction loss and recurrence of ulcer.

Although a stable sitting balance was obtained after surgery in the present case, unfortunately an ulcer in the perineal area developed, possibly due to changes in the pressure distribution caused by the realignment of the spinal column. This complication should be considered as possible complication when kyphectomy is indicated in adult patients with myelomeningocele.

## Conclusion

We experienced a rare adult case of severe kyphosis associated with myelomeningocele that developed pyogenic spondylitis at the apex of kyphosis. Kyphectomy from L1 to L3 followed by segmental pedicle screw fixation was performed to prevent recurrence of the ulcer. Development of sagittal imbalance could be avoided by approximating the ventral wall of the T12 vertebral body and cranial endplate of the L4 vertebra body.

## Consent

Written informed consent was obtained from the patient for publication of this case report and any accompanying images. A copy of the written consent is available for review by the Editor-in-Chief of this journal.

## Competing interests

The authors declare that they have no competing interests.

## Authors' contributions

KY, KW and MM made substantial contributions to the conception and design, and the acquisition, analysis, and interpretation of data. They were also involved in drafting and revising the manuscript. YT and KC contributed to the conception and design, performed critical revision of the manuscript and gave final approval of the version to be published.
